# Experimentally induced myopia and myopic astigmatism alter retinal electrophysiology in chickens

**DOI:** 10.1038/s41598-022-25075-8

**Published:** 2022-12-07

**Authors:** Sonal Aswin Vyas, Yamunadevi Lakshmanan, Henry Ho-lung Chan, Tsz-wing Leung, Chea-su Kee

**Affiliations:** 1 School of Optometry, The Hong Kong Polytechnic University, Hong Kong, SAR China; 2 Laboratory of Experimental Optometry (Neuroscience), School of Optometry, The Hong Kong Polytechnic University, Hong Kong, SAR China; 3 Centre for Eye and Vision Research (CEVR), 17W Hong Kong Science Park, Hong Kong, China; 4 Research Centre for SHARP Vision (RCSV), The Hong Kong Polytechnic University, Kowloon, Hong Kong

**Keywords:** Refractive errors, Animal disease models

## Abstract

Myopia (or “short-sightedness”) and astigmatism are major causes of visual impairment worldwide. Significant amounts of astigmatism are frequently observed in infants and have been associated with myopia development. Although it is well established that both myopia and astigmatism are associated with ocular structural changes from anterior to posterior segments, very little is known on how these refractive errors alter retinal functions. This study investigated the effects of experimentally induced myopia and myopic-astigmatism on retinal electrophysiology by using an image-guided, multifocal global flash stimulation in chickens, a widely used animal model for refractive error development. Myopia and myopic-astigmatism were experimentally induced, respectively, by wearing spherical (− 10 D, n = 12) and sphero-cylindrical lenses (− 6.00 DS/− 8.00 DCx90: Hyperopic With-The Rule, H-WTR, n = 15; − 6.00 DS/− 8.00 DCx180: Hyperopic Against-The-Rule, H-ATR, n = 11) monocularly for a week (post-hatching day 5 to 12). An aged-matched control group without any lens treatment provided normal data (n = 12). Multifocal electrophysiological results revealed significant regional variation in the amplitude of induced component (IC) (central greater than peripheral; both p < 0.05) in the normal and H-ATR groups, but not in the – 10 D and H-WTR groups. Most importantly, for the first time, our results showed that both H-WTR and H-ATR groups exhibited a significantly longer implicit time of the inner retinal response at the central region when compared to the normal and – 10 D groups, highlighting a significant role of astigmatism in retinal physiology.

## Introduction

Myopia and astigmatism are among the most common causes of vision impairment, particularly in children aged 5 to 15 years^1^. Longitudinal studies in children have shown that the presence in early years of a specific astigmatic subtype, ATR astigmatism, in which the horizontal meridian has stronger refractive power than vertical, could promote myopia development^2,3^ and progression^4^. In addition, myopia and astigmatism frequently co-exist in humans and animal models with abnormal refractive development^5^, suggesting a potential interaction of these two refractive errors in modulating early eye growth. Cumulative evidence from human and animal studies have demonstrated that postnatal refractive development is regulated by visual signals^6^, but whether and how the presence of myopia and myopic-astigmatism influence the retinal signaling pathway remains largely unknown.

The application of retinal electrophysiological measurement on human myopic adults revealed altered retinal function that was attributed to the long-term effects of myopia^7–10^. Recently, a modified multifocal electroretinogram (mfERG) paradigm^11,12^ (Global-Flash Multifocal Electroretinogram; MOFO mfERG) has been employed to examine both the outer and inner retinal responses simultaneously in myopic eyes^13^. The results showed a reduction of inner retinal function during myopia development in children^13^, indicating altered neuronal activities in the inner retinal layer during refractive error development. Using the same paradigm, Li et al. found that emmetropic children who had a reduced inner retinal function in the central region were more likely to later develop myopia^14^, indicating the importance of measuring electrophysiological responses at early age.

The availability of experimental animal models allows assessment of structural and functional changes related to myopia progression under well-controlled conditions. While structural changes (e.g., axial length, choroidal thickness) have been consistently observed in myopia animal models^6^, retinal functional changes were reported in only a few studies^15,16^. Fujikado et al., using full-field flash electroretinogram (ffERG), compared the retinal function of myopic chicks induced by form deprivation and lens induction paradigms^15^. They found that while a- and b-wave amplitudes were similar in chicks reared under these two paradigms, the oscillatory potentials (Ops), reflecting the inner retinal activity, were significantly reduced only in the form-deprived chicks, suggesting differential impacts of rearing paradigms on neural signaling pathways. More recently, Schmid et al. compared mfERG responses between control and form-deprived chicks at different time points and found that both amplitude and implicit time of N1P1 responses in form-deprived chicks were different from those of controls^16^. Thus, both human and animal studies have shown altered inner retinal functions in myopic eyes, but whether and how these functions are also affected by myopic-astigmatic development have not been investigated.

Using sphero-cylindrical lenses to impose hyperopic astigmatism, which is commonly found in the clinic, we recently reported the differential effects of astigmatic subtypes on myopia progression^17^. In the current study, it was investigated whether and how induced myopic-astigmatism influenced retinal functions, using a global flash mfERG protocol, assisted by a confocal scanning laser ophthalmoscope (cSLO). The combination of mfERG and cSLO paradigm allowed assessment of retinal functions at different regions (central vs. periphery) and layers (outer vs. inner). The results showed that myopic-astigmatic eyes had altered inner retina responses in the central region.

## Results

### Effects of lens treatments on refractive and axial parameters

#### Comparison of normal and lens treatment groups

**Figure 1:**
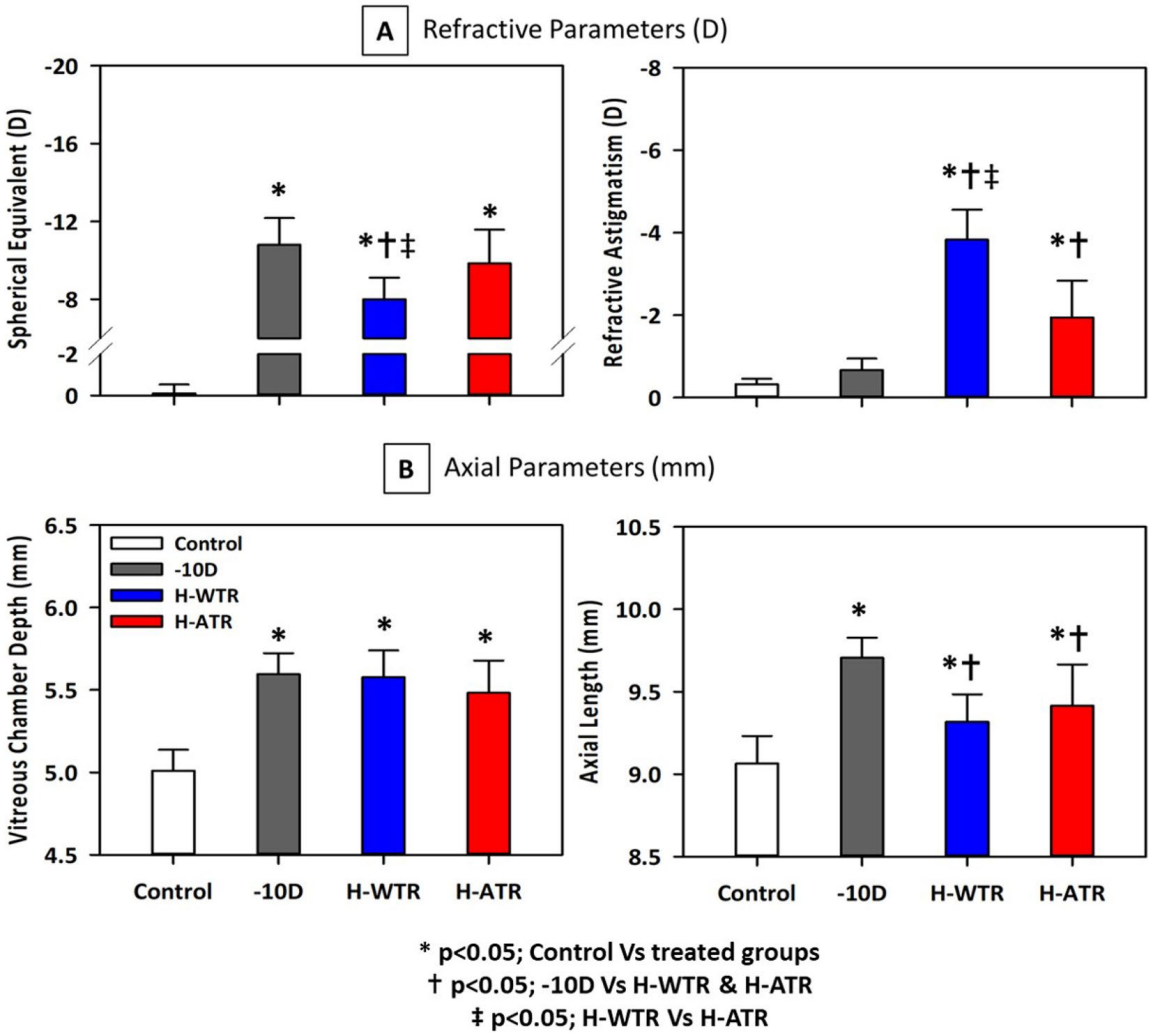
Effect of visual manipulations on refractive (**a**) and axial (**b**) parameters. Comparisons of (**a**) refractive (spherical equivalent and refractive astigmatism) and (**b**) axial (vitreous chamber depth and axial length) parameters across four groups of birds were performed using one-way ANOVA with Bonferroni’s pairwise post-hoc tests. The level of significance is indicated as follows: *p < 0.05, comparisons between normal and treated groups (− 10 D, H-WTR and H-ATR); ^†^p < 0.05, comparisons between – 10 D and the two sphero-cylindrical lenses treated groups (H-WTR and H-ATR); ^‡^p < 0.05, comparisons between two sphero-cylindrical lenses treated groups (H-WTR and H-ATR). When compared to normal group, all three treatment groups were found to have significantly higher myopic errors, increased vitreous chamber depths and axial lengths. H-WTR group developed less myopic error but more refractive astigmatism when compared to – 10 D and H-ATR groups.

Lens treatments induced significant changes in ocular refractive and axial parameters (Fig. 1 and Table 1). Compared to the normal group, all lens treatment groups (− 10 D, H-WTR and H-ATR) developed higher magnitudes of myopia (One-way ANOVA with Bonferroni’s post hoc tests, all p < 0.001; Fig. 1a), deeper vitreous chamber depth (VCD) (One-way ANOVA with Bonferroni’s post hoc tests, all p < 0.001; Fig. 1b), and longer axial length (all p < 0.001; Fig. 1b). In addition, compared to the normal group, both sphero-cylindrical lenses treated groups (H-WTR and H-ATR) developed higher refractive astigmatism (One-way ANOVA with Bonferroni’s post hoc tests, both p < 0.001; Fig. 1b) and J0 astigmatic components (both p < 0.01; Table 1). The H-WTR group also had a higher J45 astigmatic component (p < 0.001). In contrast, both – 10 D and H-ATR treated groups developed a deeper anterior chamber depth (ACD) (both p < 0.001), and the H-ATR group had a thicker scleral thickness (p < 0.01) than the normal group.

**Table 1:** Effects of the visual manipulations on refractive and axial parameters.

Groups (number of birds)	Normal (12)	LIM (12)	H-WTR (15)	H-ATR (11)
**Refractive parameters (D)**				
M	− 0.11 ± 0.12	− 10.81 ± 0.40*	− 8.00 ± 0.29*^†‡^	− 10.82 ± 0.50*
RA	− 0.32 ± 0.04	− 0.67 ± 0.08	− 3.83 ± 0.19*^†‡^	− 1.95 ± 0.27*^†^
J0	− 0.15 ± 0.02	− 0.31 ± 0.04	− 1.73 ± 0.08*^†‡^	0.34 ± 0.18*^†^
J45	0.01 ± 0.02	− 0.06 ± 0.03	0.75 ± 0.11*^†‡^	− 0.43 ± 0.22
**Axial parameters (mm)**				
CCT (µm)	175.8 ± 2.58	174.3 ± 2.16	179.9 ± 2.49	173.1 ± 4.61
ACD	1.23 ± 0.02	1.36 ± 0.01*	1.29 ± 0.02^‡^	1.40 ± 0.02*
LT	2.06 ± 0.03	2.03 ± 0.02	2.03 ± 0.02	2.02 ± 0.02
VCD	5.01 ± 0.04	5.60 ± 0.04*	5.58 ± 0.04*	5.48 ± 0.06*
RT	0.24 ± 0.01	0.24 ± 0.01	0.24 ± 0.00	0.25 ± 0.01
CT	0.22 ± 0.02	0.19 ± 0.01	0.18 ± 0.01	0.22 ± 0.01
ST	0.12 ± 0.00	0.12 ± 0.01	0.12 ± 0.00^‡^	0.14 ± 0.01*^†^
AXL	9.06 ± 0.05	9.71 ± 0.04*	9.32 ± 0.04*^†^	9.41 ± 0.08*^†^

Comparison of refractive and axial parameters in the treated eyes across the four groups were performed using One-way ANOVA with Bonferroni’s post-hoc tests. In addition, independent t-tests were performed to compare between the two sphero-cylindrical lens-wear groups (H-WTR and H-ATR). The level of significance is represented as follows: *p < 0.05, comparisons between the Normal group and the 3 lens treated groups (− 10 D, H-WTR and H-ATR); ^†^p < 0.05, comparisons between the – 10 D group and the two sphero-cylindrical lenses treated groups (H-WTR and H-ATR); ^‡^p < 0.05, comparisons between the two sphero-cylindrical lens-wear groups (H-WTR and H-ATR). *M* spherical equivalent, *RA* refractive astigmatism, *J0* refractive J0, *J45* refractive J45, *CCT* central corneal thickness, *ACD* anterior chamber depth, *LT* lens thickness, *VCD* vitreous chamber depth, *RT* retinal thickness, *CT* choroidal thickness, *ST* scleral thickness, *AXL* axial length.

#### Comparison of lens treatment groups

Compared to the – 10 D group, both sphero-cylindrical lens-wear groups (H-WTR and H-ATR) developed higher refractive astigmatism (One-way ANOVA with Bonferroni’s post hoc tests, both p < 0.001), higher J0 astigmatic component of different signs (One-way ANOVA with Bonferroni’s post hoc tests, both p < 0.001), and shorter axial length (One-way ANOVA with Bonferroni’s post hoc tests, both p < 0.01). In addition, compared to the – 10 D group, the H-WTR group had a higher J45 astigmatic component (p < 0.001), and H-ATR had greater scleral thickness (p < 0.01). Comparison of the two sphero-cylindrical lens-wear groups showed that the H-WTR group developed less myopia, higher refractive astigmatism, significantly different J0 and J45 astigmatic components, a shallower anterior chamber, and thinner scleral thickness than the H-ATR group (One-way ANOVA with Bonferroni’s post hoc tests, all p < 0.01). The H-WTR group also developed less myopia compared to – 10 D (p < 0.001) and H-ATR (p < 0.001) groups. No other parameters showed statistically significant differences.

### Effects of lens treatments on retinal electrophysiological responses

**Figure 2:**
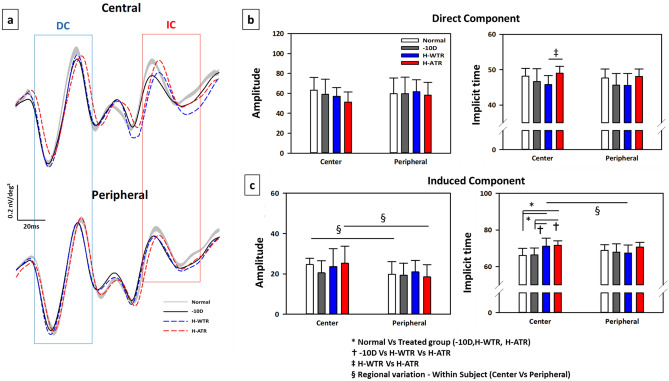
Effects of lens treatment and retinal region on ERG responses. (**a**) Averaged central and peripheral MOFO waveforms from normal (Grey) and three lens treatment groups (Black: − 10 D, n = 12; Blue: H-WTR, n = 15; and Red: H-ATR, n = 11). Note that the waveforms are normalized at the starting point for easy comparison. Boundaries of grey area mark the upper and lower limits of 95% CI of the mean waveform from the normal eyes (n = 12). The blue and red boxes enclose the direct (DC) and induced (IC) components, respectively. Bar graphs shows the amplitude (left) and implicit time (right) of direct (**b**) and induced (**c**) components from the normal and the three lens treatment groups. A mixed-model ANOVA was used to compare the differences in ERG responses (amplitude and implicit time) within groups (central versus peripheral) and between groups (across 4 groups: − 10 D, H-WTR & H-ATR) with Bonferroni corrections. The level of significance is indicated as follows: *p < 0.05, comparisons between groups (normal vs three treatment groups); ^†^p < 0.05, comparisons between – 10 D and two sphero-cylindrical lenses treated groups; ^‡^p < 0.05, comparisons between two sphero-cylindrical lenses treated groups (H-WTR and H-ATR); and ^§^p < 0.05, comparisons within groups (central vs peripheral) between regions. Compared to normal and -10 D group, both sphero-cylindrical lens-wear groups (H-WTR and H-ATR) had delayed induced component (longer IC implicit time) in the central region. Both normal and H-ATR groups had significantly higher IC amplitude in the central than peripheral.

The waveform of MOFO retinal responses consists of two components: a direct component (DC) which reflects outer retinal activity, and an induced component (IC), which predominantly reflects the neural activity of the inner retinal neurons (see Discussion section for more details). The effects of lens treatment (normal vs. treated groups) and retinal region (central vs. peripheral) on the amplitude and implicit time of MOFO retinal responses are presented in Fig. 2 and Table 2. Figure 2a superimposes the averaged MOFO waveforms from the normal (Grey area) and three lens treatment groups (− 10 D: Black solid line; H-WTR: Blue dash line; and H-ATR: Red dash line) collected at the central (Top) and peripheral (Bottom) retinal regions. The grey area of the normal waveform represents the 95% confidence interval of the mean. Mixed model ANOVAs (between groups: across 4 groups; within groups: central vs peripheral) showed that lens treatment had a significant effect on both direct component (DC) and induced component (IC) implicit times (p < 0.05), whilst the retinal region had a significant effect on DC implicit times (p < 0.05) and IC amplitude (p < 0.001). In addition, there was a significant interaction effect (lens treatment x retinal region) on IC implicit time (p < 0.01). For DC, the amplitude was not significantly different between any of the groups (all p > 0.05), but the implicit time in the H-ATR group was significantly longer than that of the H-WTR group (p < 0.05). For IC, both normal and H-ATR groups had higher amplitude at the central than the peripheral region (p < 0.05), but this regional variation was not observed in the – 10 D and H-WTR groups (p > 0.05). Lastly, the IC implicit time at the central region was significantly delayed (longer) in the two sphero-cylindrical lens-wear groups compared to the normal (both p < 0.01) and – 10 D (both p < 0.01) groups. There were no other significant effects on retinal electrophysiological function.

**Table 2:** Effects of visual manipulations on the amplitude and implicit time of MOFO responses.

MOFO Responses		Direct component (DC)				Induced component (IC)			
Group (number of birds)		Normal (12)	− 10 D (12)	H-WTR (15)	H-ATR (11)	Normal (12)	−10 D (12)	H-WTR (15)	H-ATR (11)
**Amplitude (nV/deg**^**2**^**)**	C	63.16 ± 12.74	59.04 ± 15.28	57.07 ± 8.66	51.30 ± 10.17	24.64 ± 3.17^§^	20.58 ± 5.94	23.71 ± 8.76	25.25 ± 8.54^§^
	P	59.67 ± 15.88	59.77 ± 16.42	61.66 ± 12.10	58.24 ± 12.86	19.84 ± 6.24	19.34 ± 5.94	20.99 ± 5.75	18.55 ± 6.07
**Implicit time (ms)**	C	48.18 ± 2.18	46.67 ± 3.58	45.77 ± 2.59^‡^	49.03 ± 1.96	66.08 ± 3.84	66.33 ± 3.86	71.13 ± 4.39*†^§^	71.62 ± 2.40*†
	P	47.63 ± 2.58	45.68 ± 3.23	45.58 ± 3.37	48.13 ± 2.09	68.77 ± 3.08	67.91 ± 4.43	67.37 ± 4.38	70.57 ± 2.75

Comparisons of amplitude and implicit time of the direct (DC) and induced (IC) components across the four groups of birds. Note that the amplitudes of direct component (DC) and induced component (IC) were calculated from peak-to-peak, whereas the implicit times of DC and IC responses were calculated from the onset of multifocal flash and global flash, respectively, as illustrated in the Fig. 4d. A mixed-modal ANOVA was used to compare the differences in MOFO responses (amplitude and implicit time) between groups (Normal, − 10 D, H-WTR and H-ATR groups) and within groups (central [C] vs peripheral [P]). The level of significance is indicated as follows: *p < 0.05, comparisons between groups (normal vs three treatment groups); ^†^p < 0.05, comparisons between – 10 D and two sphero-cylindrical lenses treated groups; ^‡^p < 0.05, comparisons between two sphero-cylindrical lenses treated groups (H-WTR and H-ATR) and ^§^p < 0.05, comparisons within groups (central vs peripheral) between regions.

### Correlations between refractive and axial parameters with MOFO responses

**Figure 3:**
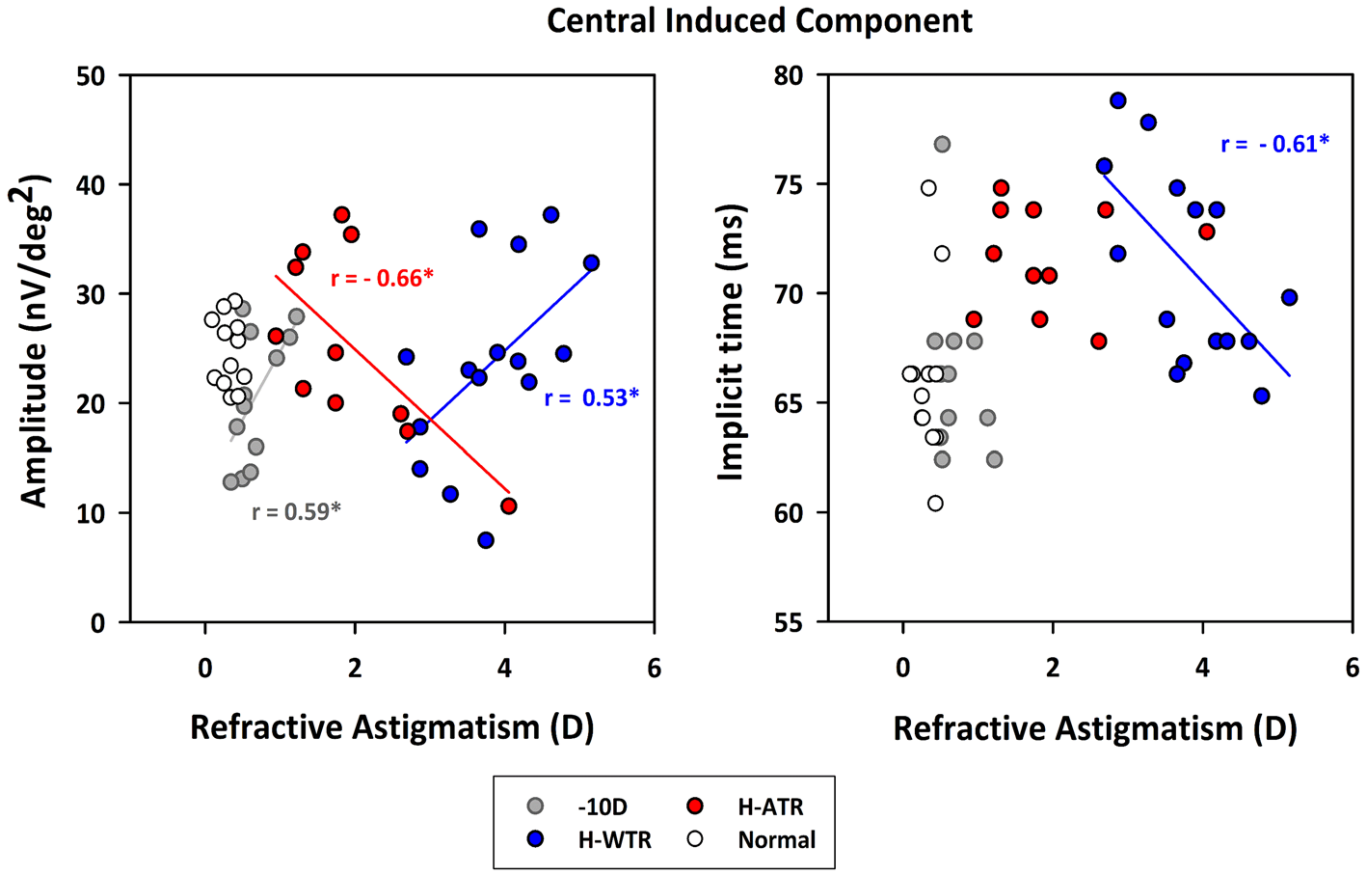
Correlations between refractive astigmatism with amplitude (Left) and implicit time (Right) of the central induced component (IC). Refractive astigmatism is plotted against the amplitude (left) and implicit time (right) of the central IC responses in normal and treated eyes with spherical (− 10 D) or the two sphero-cylindrical lenses (H-WTR and H-ATR). Different colored symbols/lines represent different groups as shown in the legends. Only Pearson’s r with statistical significance is shown. The levels of significance are represented by asterisk: p < 0.05*.

Table 3 summarizes the significant correlations between the refractive and axial parameters with MOFO responses in the different treatment groups. For the normal group, the vitreous chamber depth was significantly correlated with the central IC implicit time (Pearson’s r = 0.60; p < 0.05). Whilst the – 10 D group only showed a significant correlation between refractive astigmatism with the central IC amplitude (Pearson’s r = 0.59, p < 0.05), both sphero-cylindrical lens-wear groups showed multiple correlations between both refractive and axial components with individual MOFO responses (Pearson’s r range = 0.53 to − 0.66, all p < 0.05; see Table 3 for details). Most of these correlations were found with MOFO responses in the central region, of which the H-WTR group MOFO responses were correlated with the highest number of refractive and axial parameters. Among these parameters, refractive astigmatism was the only one that was associated with MOFO central IC for all three lens treatment groups. As shown in Fig. 3, both – 10 D and H-WTR groups displayed positive correlations of refractive astigmatism with central IC; in contrast, the H-ATR group showed a negative correlation between these two parameters. In addition, a higher refractive astigmatism in H-WTR was associated with a shorter central IC implicit time (Fig. 3 and Table 3). For the two astigmatic components, the J0 component of the H-WTR group was significantly correlated with the implicit time of both DC (r = − 0.54; p < 0.05) and IC (r = 0.80; p < 0.05) responses in the central region, whereas the J45 component of H-ATR group was correlated with the central IC amplitude (r = − 0.66, p < 0.05; Table 3). In contrast, both sphero-cylindrical lens-wear groups showed a positive correlation between central corneal thickness and central IC amplitude, and the H-WTR group also showed significant correlations between individual MOFO components and retinal and choroidal thickness (Table 3).

**Table 3:** Correlations between refractive and axial parameters with retinal responses.

Group (number of birds)		Normal (12)	− 10 D (12)	H-WTR (15)	H-ATR (11)
**Refractive components**	M				Peripheral DC Implicit Time (-0.64)
	RA		Central IC amplitude (0.59)	Central IC amplitude (0.53) Central IC implicit time (− 0.61)	Central IC Amplitude (-0.66)
	J0			Central DC implicit time (− 0.54) Central IC implicit time (0.80)	
	J45				Central IC Amplitude (0.66)
**Axial components**	CCT			Central IC amplitude (0.55)	Central IC Amplitude (0.65)
	VCD	Central IC implicit time (0.60)			
	RT			Central DC implicit time (0.63)	
	CT			Peripheral IC amplitude (− 0.56)	

Significant correlations were found between refractive (M, spherical equivalent; RA, refractive astigmatism; J0, refractive J0; and J45, refractive J45) or axial (CCT, Central corneal thickness; RT, retinal thickness; and CT, choroidal thickness) parameters with the amplitude and implicit time of DC and IC responses. Only statistically significant correlations with the MOFO responses are presented in the table.

## Discussion

The key findings in this study were: (1) normal and H-ATR groups showed a higher inner retinal response (IC) in the central than the peripheral region, but this regional variation was not observed in either the – 10 D or H-WTR groups; (2) compared to the normal and – 10 D groups, the sphero-cylindrical lens-wear groups showed delayed inner retinal responses in the central region; and (3) in all lens-treatment groups, refractive astigmatism was significantly associated with the amplitude of inner retinal responses in the central region.

This study incorporated two new approaches to measurement of the regional retinal function in chickens: First, the use of an image-guided technique to project and track the visual stimuli on a designated retinal area throughout the testing period, overcoming the potential variations due to small saccadic eye movements^18^ and the misalignment of testing stimuli with the retinal region of interest^19^ when recording mfERG responses in anaesthetized animals, thereby providing a reliable regional retinal responses^20.^ Second, in contrast to previous studies, which used a standard mfERG protocol, a variant of the mfERG (MOFO) paradigm^13,14^ was used to assess the outer and inner retinal responses^11,21^. The use of these two approaches allowed the determination of the impacts of myopic and myopic-astigmatic eye growth on electro-retinal activities similar to those reported in human myopes^22^. The results not only confirm and extend the current understanding of how abnormal refractive development may affect retinal electrophysiological responses, but also demonstrate the feasibility of using chicken model to study the role of retinal signaling pathway in myopes and myopic-astigmats.

The myopic chicks induced by – 10 D lens-wear in this study showed a reduction of inner retinal responses (IC) in the central region. While the normal birds showed a higher IC amplitude in the central than the peripheral region (approximately 1.2 times; Fig. 2c), this regional difference in IC amplitude was lost in the – 10 D group: the central IC amplitude of these myopic birds was only as high as the peripheral IC amplitude of normal birds (Fig. 2a,c), indicating an intact outer retinal layer (normal DC response), but an affected inner retinal layer (altered IC response). Support for this interpretation comes from pharmacological dissection studies, which reported the cellular origin of MOFO responses in pigs^21^ and chicks^23^: The DC response is primarily attributable to the photoreceptors and bipolar cells, whereas the IC originates from cells in the inner retinal layer. Results of this study showing altered inner retinal function in myopic chick eyes are consistent with those reported by Fujikado et al.^15^. Specifically, these researchers compared the retinal function of form-deprived (FDM) and lens-induced myopic (LIM) chickens using full-field electroretinogram (ffERG) and found that FDM chicks (− 23 D) had reduced oscillatory potentials with intact a- and b-wave amplitudes than LIM chicks (about – 18 D). Although they did not compare the retinal function of LIM chicks to control birds, the data presented in their study (see Table 1 in Fujikado et al.^15^) revealed a tendency of reduced amplitude of oscillatory potentials in the LIM chicks (op1: 0.75 ± 0.15 and op2: 0.81 ± 0.13) when compared to the control chicks (op1: 0.82 ± 0.15 and op2: 0.84 ± 0.18). These results indicate that the myopia induced by minus lens-wear was associated with altered oscillatory potentials generated by the inner retinal cells. In this respect, the current study provides direct evidence of region-specific inner retinal dysfunction in lens-induced myopic chicks. There is ample evidence of the involvement of the inner retinal layer during myopia development^6,24–30^. First, decreased retinal dopamine levels were reported in chicken developing form deprivation myopia^26^. This reduced dopamine level has been hypothesized to reduce the number of inhibitory synapses of amacrine and ganglion cells, resulting in reduced inner retinal activity^15^. Second, the bi-directional changes of ZENK expression^31^ in retinal glucagon-amacrine cells in response to hyperopic and myopia defocus suggest the involvement of inner retinal cells in the signaling pathway that modulates eye growth. Third, myopic chicken eyes had significantly thinner retinal nerve fiber and ganglion cells layers after 5 days of FDM and LIM compared to control birds^32^. Fourth, electrophysiological studies in humans showed reduced inner retinal activity associated with myopia progression^33,34^ and prior to the development of myopia^14^ in children. Taken together, all these results support the hypothesis that myopia alters inner retinal function. However, it is important to consider the anatomical difference in retinal organization between humans and chicks. Although the retina of chicks has a similar retinal organization to that of humans, they have some key structural differences^6,35^. Unlike humans, the chick’s retina has an afoveate region, commonly known as the “area centralis”, located approximately 2 mm away from the dorsal edge of the optic disc^36^. Similar to the human fovea, this region is a rod-free zone with a higher density of cones^37^ and better visual acuity^38^. The remainder of the chick retina has an organization similar to that of humans with three layers of nuclei^35^. In terms of retinal electrophysiological responses, Ostrin et al. showed that PERG responses were not affected despite the loss of retinal ganglion cells in chicks with optic nerve section (ONS), suggesting that retinal ganglion cells do not contribute to PERG in chicks^39^. These results await a systematic follow-up using pharmacological dissection to study the neuronal origins of MOFO responses.

Compared to the normal and -10D groups, both sphero-cylindrical lenses groups showed delayed IC responses in the central region (Fig. 2c, Table 2). As illustrated in the MOFO IC waveforms of the central region in Fig. 2a (top, red box), both H-WTR (blue) and H-ATR (red) had increased latency of approximately 7.8% when compared to the normal (grey) and – 10 D (black) groups. Compared to the DC waveforms in the central region (top, blue box), the IC waveforms became more variable across groups even before their leading edges (left border of red box). Thus, what was observed in IC implicit time using the conventional approach might have captured only one operational difference in retinal responses between the treatment groups. When mfERG responses were compared across three time points in a group of chicks made myopic by full-field form deprivation, noticeable timing differences in mfERG waveform were also noted^16^. It should be noted that in addition to an elongated eyeball in our sphero-cylindrical lens treated chicks, they also developed a significantly higher magnitude of astigmatism compared to -10D lens (in particular the WTR treated group, Table 1), in agreement with the refractive and axial changes using sphero-cylindrical lenses with different magnitudes and axes in a separate study^17^. Because earlier studies usually used spherical equivalent to represent refractive change and changes in J0 and J45 astigmatic components were not available, it is difficult to directly compare the results between Schmid et al. (2013) with the current study. On the other hand, results from monkeys have suggested that when the mfERG amplitude is normal, but the implicit time is increased, this might be due to altered pre-synaptic transmission or inner plexiform activity^40^. At this stage, our findings suggest that myopic-astigmatic development could influence the central retinal responses, but further investigation is required to understand the origin of this altered waveform.

A novel finding from the current study is the relationship between the magnitudes of refractive astigmatism and central IC (Fig. 3). Although astigmatism has been linked to visual functions such as contrast sensitivity^41^ and resolution acuity^42^, studies investigating the electrophysiological responses have usually only included subjects with a low degree of astigmatism^13,14^. To the best of our knowledge, only one study reported a significant association between high astigmatism (> 1.5 D) and ERG abnormalities in human ametropes (high myopia to high hyperopia)^43^. In the current study, a higher refractive astigmatism was associated with a decreased central IC response in the H-ATR group, but with an increased central IC response in the H-WTR group (Fig. 3). In addition, H-ATR showed a reduced IC amplitude at the peripheral region than the central region, but this regional difference was not observed in the H-WTR group. Furthermore, the H-ATR group showed a delayed DC implicit time in the central region when compared to the H-WTR group. One possible explanation for this differential effect of astigmatism on retinal responses may be related to the orientation sensitivity of the visual system^44^. Previous studies in both animal^45–47^ and human visual systems^48^ have reported that the retinal ganglion cells are orientation sensitive; in essence, the visual system or function is tuned to horizontal-vertical orientation over oblique orientation^44,48–51^, an effect termed “oblique effect”^49^. In the current study, the astigmatism induced by the sphero-cylindrical lenses had axis orientations clustered near 90° (H-WTR) and 180° (H-ATR) axes (Table 1, Supplementary Fig. 2). Furthermore, the H-WTR group developed significantly higher magnitudes of refractive astigmatism than the H-ATR group (Table 1), only three H-ATR birds developed astigmatism ≥ 2D with axes oriented obliquely (see Supplementary Fig. 2). It may be speculated that these astigmatic properties may contribute to the differential central IC amplitudes between the H-WTR and H-ATR groups; while the spherical-equivalent refractive error was corrected by the instrument during ERG recording (see method section), those birds with a higher astigmatism (after corneal astigmatism was partially neutralized by the compression of the probe) of 90°/180° axis showed a higher IC amplitude, whereas a few birds in the H-ATR group with a higher astigmatism, but oblique axis showed lower IC amplitude (see Fig. 3), resulting in a different relationship between astigmatism and IC amplitude in the two groups of birds. This “oblique effect” hypothesis is further supported by the observation that the J45 astigmatic component (an indicator of oblique astigmatism) in the H-ATR group was significantly correlated with the central IC amplitude (r = 0.66, P = 0.03; see Table 3), indicating a reduced IC amplitude with obliquely oriented astigmatism (see Supplementary Fig. 2). In humans, the amplitude of MOFO responses in six peripheral retina areas (15° eccentricity, 60° apart radially) was positively correlated with local hyperopic errors, but the MOFO responses were neither correlated with the local astigmatic component nor with the optically imposed astigmatism^52^. In our study, all the MOFO responses that showed significant correlations with astigmatic components were found in the central region (see Table 3, RA, J0 & J45). Taken together, our results suggest a significant role of astigmatic properties in signal processing within the area centralis, at least at the time point examined in the study.

## Limitations of current study

To the best of our knowledge, this is the first study to investigate the influence of myopia and myopic-astigmatism on retinal electrophysiological responses in chicks. However, three limitations of this study do caution its interpretation. First, the influence of the astigmatism was assessed only after 7 days of treatment. Although 7 days of lens treatment (P5–P12) induced significant refractive and axial changes compared to the age-matched normal group, this cross-sectional design only captured the noticeable changes in retinal responses at one time point (P12). An earlier study reported retinal electrophysiological changes after 2 h of form deprivation, suggesting a rapid change in retinal responses^16^. The retinal electrophysiological changes reported in the current study may only represent the consequence of abnormal refractive development. While it provides us with a basis for use of the mfERG in the chick model for investigations of retinal processes in myopic and myopic-astigmatic eye growth, extrapolating this result to other time points or other species awaits further investigations. Second, the MOFO mfERG responses were recorded using the 61-hexagon stimuli, covering approximately 34° horizontal and 30° vertical visual fields. In chicks, the area centralis is approximately 3 mm in diameter (~ 23.04°)^35^. On this basis, the mfERG responses obtained in our study represented mainly the central (19° and 17° horizontal and vertical visual fields) and mid-peripheral retina (19° to 34° and 17° to 30° horizontal and vertical visual fields). In order to understand the role of the peripheral retinal region during abnormal refractive development, future experiments may consider using 103-hexagon stimuli to measure the responses from a wider retinal area. Third, to explore the potential effects of uncorrected astigmatic defocus on mfERG responses, the image-guided SLO-mfERG provided good control of delivering the stimuli with optical correction for spherical-equivalent refractive error but not for cylindrical correction. Although the induced refractive astigmatism (− 3.83 ± 0.19D) might have been partially neutralized when the probe pressed on the cornea (thereby removing some corneal astigmatism contributing to the total refractive astigmatism), it was not possible to rule out the potential optical effect arising from residual internal astigmatism affecting the mfERG responses. In this regard, a previous human study showed that astigmatic defocus up to 3D did not significantly alter the amplitude or implicit time of the mfERG responses^53^. Given the evidence that about 40% of the induced refractive astigmatism is contributed by corneal astigmatism (Supplementary Fig. 2), it is possible that the mfERG responses we observed were partially affected by the uncorrected residual astigmatism. Better optical control of the stimuli presentation is needed to confirm these results.

## Conclusion

In summary, both myopic and myopic-astigmatic eye growth altered retinal responses in the central region. The differential effects of ATR and WTR astigmatism on eye growth and retinal functions underscore the significant role of astigmatism in refractive development. Further studies are needed to investigate the retinal neuronal pathways and cell types involved in this process.

## Materials and methods

### Animals

Fifty white Leghorn chicks (*Gallus gallus domesticus*) were housed in a 12:12 light/dark cycle (on at 8 AM, 150 lx) at room temperature (22 °C). Food and water were available ad libitum. Animal care and experimental procedures were undertaken in accordance with the ARVO statement for the Use of Animal in Ophthalmic and Vision Research and ARRIVE guidelines (Animal Research: Reporting of In Vivo Experiments). The study was approved by the Animal Subjects Ethics Sub-committee of the Hong Kong Polytechnic University (ASESC No: 16-17/86).

### Visual manipulations

On post-hatching day 5 (P5), chicks were randomly assigned to the control or three experimental groups. Table 4 summarizes the monocular treatments received by four groups of chicks starting from P5: (A) aged-matched normal birds without any treatment (normal group; n = 12); (B) negative spherical lens-wear (− 10.00 DS; n = 12); (C) sphero-cylindrical lens-wear, axis oriented vertically; with-the-rule (H-WTR: − 6.00 DS/− 8.00 DC × 90; n = 15); (D) sphero-cylindrical lens-wear, axis oriented horizontally; against-the-rule (H-ATR: − 6.00 DS/− 8.00 DC × 180; n = 11). These sphero-cylindrical lenses were chosen because they can induce the highest magnitude of refractive astigmatism at the end of the 7-day treatment period, compared to a lower power of sphero-cylindrical lenses (− 8.00 DS/− 4.00 DC)^17^. In the current study, the sphero-cylindrical lens imposed the same magnitude of spherical-equivalent refractive defocus (− 10 D) as the negative spherical lens (− 10.00 DS). The axis of sphero-cylindrical lenses was carefully oriented to simulate two types of clinical hyperopic astigmatism: with-the-rule (H-WTR: − 6.00 DS/− 8.00 DC × 90; H-WTR) and against-the-rule (H-ATR: − 6.00 DS/− 8.00 DC × 180; H-ATR)^17^.

**Table 4:**
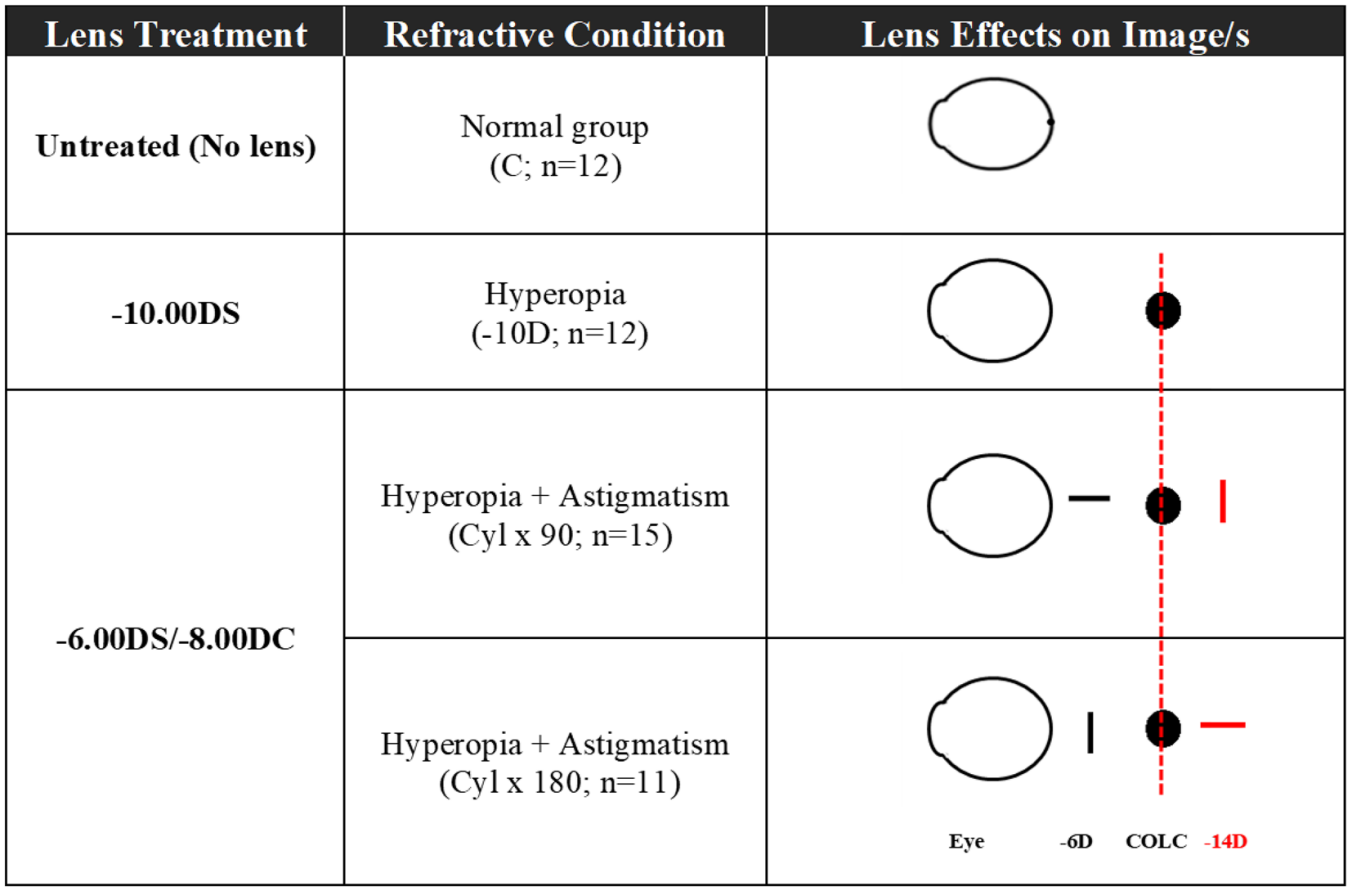
Experimental groups and their treatment conditions.

Fifty chicks were randomly assigned to four groups as follows: no treatment (Normal; n = 12), negative spherical lenses (− 10 D, n = 12), sphero-cylindrical lenses oriented vertically (H-WTR; n = 15), sphero-cylindrical lenses oriented horizontally (H-ATR; n = 11). Sphero-cylindrical lenses (− 6.00 DS/− 8.00 DC), imposing the same magnitude of spherical equivalent (hyperopic) defocus as the – 10 D group, were used to induce myopic-astigmatic eye growth. The lenses were chosen such that they impose – 8 D of astigmatism and the orientation of the sphero-cylindrical lenses was altered to simulate two common clinical forms of astigmatism: “With the rule”, WTR and “Against the rule”, ATR.

Treatment lenses (PMMA, 7.5 mm base curve, 10.8 mm diameter, 10 mm optical zone; Conforma, VA, USA) were prepared by gluing the lens to a Velcro ring using optical adhesive (Norland Products Inc., New Brunswick, NJ, USA) as described in previous studies^54,55^. On P5, the Velcro adhesive mate was glued around the feather of the right eye for each animal, and the Velcro ring with the treatment lens, aligned with the pupillary axis and palpebral fissure, was then secured firmly to this mate. The treatment period started at P5 and ended at P12 (i.e., 1 week). The left eye was left untreated. The lenses were cleaned daily and checked periodically for alignment as well as axis orientation and any debris or scratches on the lens surface. If the treatment lens was found dislocated or detached during the treatment period, the bird was excluded from further analysis.

### Measurements

At the end of the treatment period, refractive status, ocular axial dimensions, and regional retinal functions were measured using a modified Hartinger coincidence refractometer^54,55^, high-resolution A-scan ultrasonography^55^, and an image-guided mfERG system, respectively. Descriptions of the mfERG system are available in detail below whereas those for the first two methods are provided briefly here as they are available elsewhere^54,55^. In order to avoid the potential effects of the residual ultrasound gel on the cornea from affecting the data acquisition of mfERG system, A-scan ultrasonography was conducted a day earlier (P11, 11:00 am ~ 01:00 pm) than the mfERG recording. Both refractive status (09:00 am ~ 10:00 am) and mfERG (12:00–05:00 pm) were measured on P12. Considering the potential effects of isoflurane anesthesia on the eye movement and retinal functions^18^, chicks were anaesthetized using a mixture of ketamine and xylazine for the mfERG recording. Because our recent study found a small, but significant interocular effects in the fellow untreated eyes of the sphero-cylindrical lenses treated groups^17^, we only collected and compared data in the treated/right eyes of treatment and normal groups. At the end of the experiments, chicks were sacrificed by inhalation of carbon dioxide after the mfERG recordings.

### Hartinger coincidence refractometer

The refractive status of chicken eyes was measured using a modified Hartinger coincidence refractometer as described previously^54^. Briefly, under isoflurane anesthesia (1.0–1.5% in oxygen), the animal was held gently using a beak holder in an upright position on a platform. With eyelids held open using a lid retractor, three measurements of the two principle meridians were obtained and averaged using power vector analysis^56^. For data analysis, refractive errors were converted into spherical-equivalent (SE), J0, and J45 astigmatic components^56^. The two astigmatic components, J0 and J45, represented astigmatism using power vectors^56^, allowing the incorporation of the magnitude and axis of all forms of astigmatism for statistical analysis. SE, J0 and J45 astigmatic components were derived from the formulae below where $S$ is spherical power, $C$ is negative cylindrical power, and $\alpha$ is cylindrical axis.

$$SE=S+\frac{C}{2}$$

$${J}_{0}=-\frac{C}{2}\times \mathrm{cos}2\alpha$$

$${J}_{45}=-\frac{C}{2}\times \mathrm{sin}2\alpha$$

### A-scan ultrasonography

Ocular axial dimensions were measured using high-resolution A-scan ultrasonography^55,57^. The alignment of the A-scan probe with the pupillary axis was assisted by a micro-manipulator while the animal was anesthetized (isoflurane 1.0–1.5% in oxygen) and eyelids held apart using a speculum. Three recorded measurements, each consisting of fifty echograms, were averaged to obtain the individual axial components.

### Image-guided multifocal electroretinogram (mfERG)

Prior to recording, chicks were anesthetized with an intramuscular injection of ketamine (60 mg/kg) and xylazine (4 mg/kg) mixture and placed on a platform connected to a warm water bath to maintain the body temperature at 37 °C. Eyelids were held open using a speculum. A 3-mm gold ring placed on the cornea served as an active electrode. The reference and ground needle electrodes were inserted subcutaneously into the crown and on the thigh skin respectively. An impedance of less than 5 KΩ was maintained during the recording.

**Figure 4:**
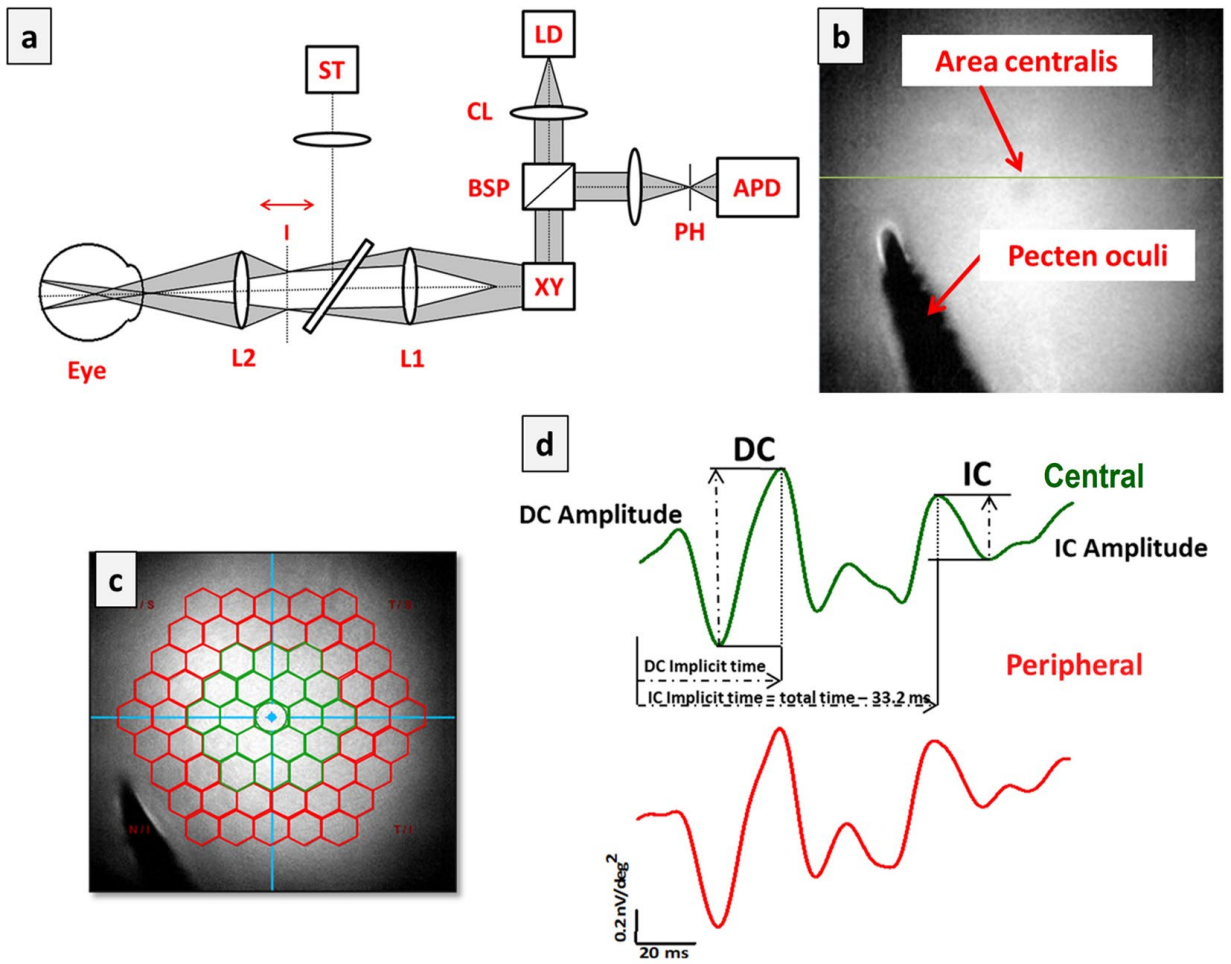
Experimental Setup, fundus image acquisition and MOFO stimulation. (**a**) Optical path of Roland Reti-Scan System, which consists of a laser point source [D], collimation lens [CL], beam splitter [BPS], scanning unit [XY], pinhole [PH], photodiode [APD], lens 1[L1] and 2 [L2], intermediate image [I], and stimulus projector [ST]. The refractive error of the eye is corrected by adjusting the intermediate image [I] using the focusing knob of the imaging system. (**b**) Fundus image of a chicken eye (12 days old) captured during OCT imaging (cSLO-OCT), also showing the location of the line scan indicated by a green line. (**c**) A fundus image superimposed by 61 unscaled hexagons (multifocal stimulation) obtained from cSLO system, showing the alignment of the center of stimuli with the area centralis. The regional responses obtained using multifocal stimulation was grouped into two regions: Central (green) and peripheral (red) regions for data analysis. (**d**) Representative mfERG waveforms obtained from the two regions (central and peripheral) in a control bird using the MOFO protocol. The measurements of the amplitude and implicit times of DC and IC components are indicated in the top panel.

Regional retinal functions were measured using an image-guided mfERG (RETIscan, Roland Consult, Wiesbaden, Germany). RETIscan is an integrated device using a confocal scanning laser ophthalmoscope (cSLO) to track the retinal region of interest when the stimulus is presented by digital light processing (DLP). Figure 4a shows the optical path of stimuli presentation by the Roland Reti-Scan system, which consists of a laser point source [LD], collimation lens [CL], beam splitter [BSP], scanning unit [XY], pinhole [PH], photodiode [APD], lenses 1[L1] and 2 [L2], intermediate image [I], and stimulus projector [ST]. Note that the spherical-equivalent refractive error of the eye was corrected by adjusting the intermediate image [I] using the focusing knob of the imaging system. Figure 4b illustrates how the area centralis was identified as the central location while mfERG waveforms were recorded for chicks. Using the OCT imaging module (Supplementary Fig. 1), the area centralis, supero-nasal to the pectin oculi, was identified^39^ and confirmed through the OCT line-scan function (green line in Fig. 4b and Supplementary Fig. 1) as a region with a relatively thicker ganglion cell layer (GCL) and a thinner nerve fiber layer (NFL) compared to the surrounding regions. During mfERG recording, the system software allowed simultaneous stimulus presentation with fundus imaging by cSLO, which ensured that the retinal region of interest was evenly illuminated and well-focused throughout the recording session. In case of significant change in retinal location due to eye movement, the recording was paused automatically by the in-built eye tracking system and resumed only after regaining the original location of the pecten by adjusting the cSLO device. Figure 4c illustrates the 61 unscaled hexagon stimuli array projected on the fundus to extract the regional retinal responses. The global flash (MOFO) mfERG paradigm^11,12^ was chosen to record both outer and inner retinal responses simultaneously. The MOFO paradigm consists of four frames in each cycle: a multifocal flash frame (M), followed by a dark frame (O), a global flash (F), and a second dark frame (O), presented according to a pseudorandom m sequence (2^9^ − 1)^11,12^. The stimulus pattern consisted of 61 unscaled hexagons with light and dark luminance of about 220 cd/m^2^ and 3 cd/m^2^ (extracted from the worksheet), respectively, covering approximately 34º horizontal and 30º vertical visual field. The contrast was set at 99%. The luminance of the hexagon-stimulus background was about 100 cd/m^2^. The examination room was dimmed (about 50 lx) and the recording time for each eye was about 8 min. The responses were band-pass filtered from 10 to 300 Hz and were amplified 100,000 times. An average of 8 cycles from each eye was used for analysis. Both the examiners were blinded from the group allocation of treated birds during the mfERG recording and data extraction.

Depending on the retinal region of interest^58,59^, the 61 hexagons can be averaged differently according to ring-wise analysis, quadrant-wise analysis, or cumulative/averaged ring responses to provide central-peripheral retinal responses. In this study, the ring-wise grouping method was chosen such that the central region corresponded to the area centralis. In chicken, the area centralis is a rod-free circular region of approximately 3 mm^35^ in diameter, corresponding to approximately 23°. Thus, the first three rings of the 61 unscaled hexagons were grouped to extract the central (Fig. 4c,R1 + R2 + R3; Green; 19° horizontal and 17° vertical visual field) and last two for the peripheral (R4 + R5; Red; 19°–34° horizontal and 17°–30° vertical visual field) retinal responses. Figure 4d shows a representative MOFO waveform retinal response recorded from a normal bird (Green: central response; Red: peripheral responses). The amplitudes of direct component (DC) and induced component (IC) were calculated from peak-to-peak, whilst the implicit times of DC and IC responses were calculated from the onset of multifocal flash and global flash^12^, respectively, as illustrated in the “central” waveform (Green).

### Statistical analysis

Data was analyzed by SPSS statistical software (IBM Inc, version 23.0.0, Illinois, USA). Comparisons of refractive and axial parameters across the four treatment groups were performed using One-way ANOVA with Bonferroni’s post-hoc tests. A mixed-model two-way ANOVA was used to compare the differences in mfERG responses (amplitude and implicit time) within groups (central vs. peripheral) and between groups (four treatment groups) with Bonferroni corrections. Pearson’s correlation analyses were performed between refractive parameters, axial parameters, and MOFO responses. Because the sphero-cylindrical lenses employed in this study showed differential treatment effects from minus spherical lenses on refractive development^17^, the correlation analyses were performed on each group separately. In all tests, the significance level was set to 95% level of confidence. Unless otherwise stated, data are expressed as mean ± standard error (SE).

## Supplementary Information

Supplementary Figures.

## Data Availability

The datasets generated during and/or analyzed during the current study are available from the corresponding author on reasonable request.
