# Supplementary material for: Experimentally induced myopia and myopic astigmatism alter retinal electrophysiology in chickens
Source: Sci Rep. 2022 Dec 7;12:21180. doi: 10.1038/s41598-022-25075-8 (PMC9729572; doi:10.1038/s41598-022-25075-8)
Supplement: Supplementary file 1 — Supplementary Figures. [file 41598_2022_25075_MOESM1_ESM.docx]

**Supplementary Materials**


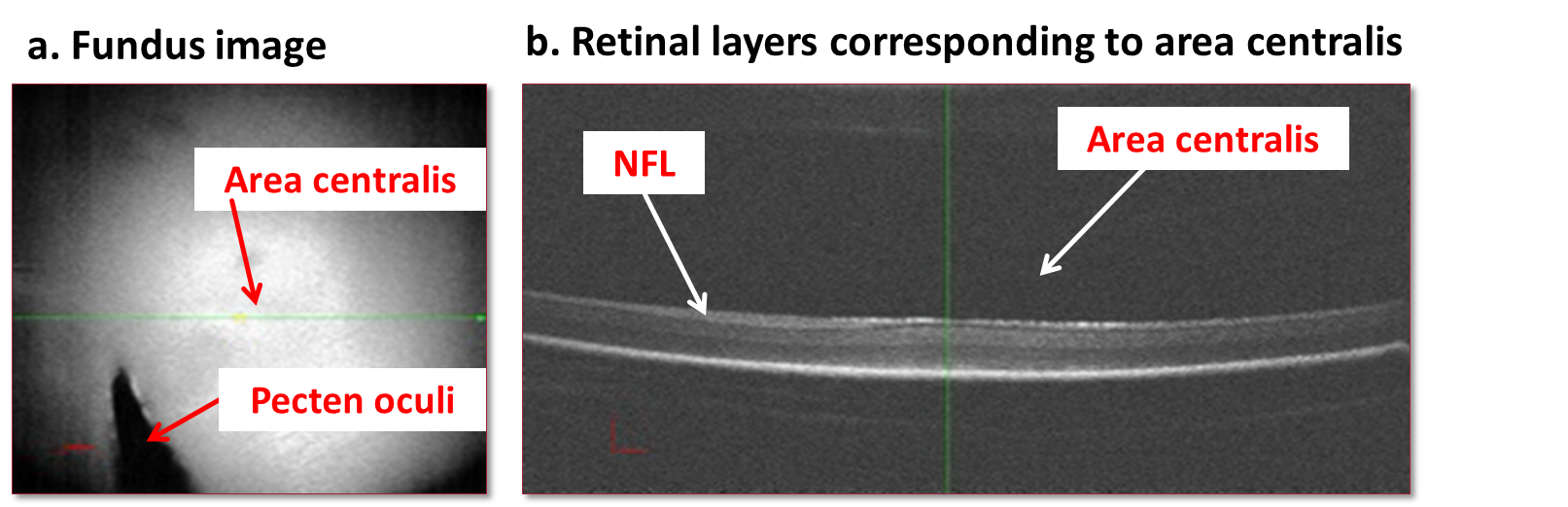


Supplementary Figure 1 Fundus image acquisition and OCT scanning.

**(a)** Fundus image of a chicken eye (12 days old) captured during OCT imaging (cSLO-OCT), also showing the location of the line scan indicated by a green line. **(b)** B-scan image of a line scan (green line) at the area centralis showing thinner nerve fiber layer (NFL) than the surrounding area.


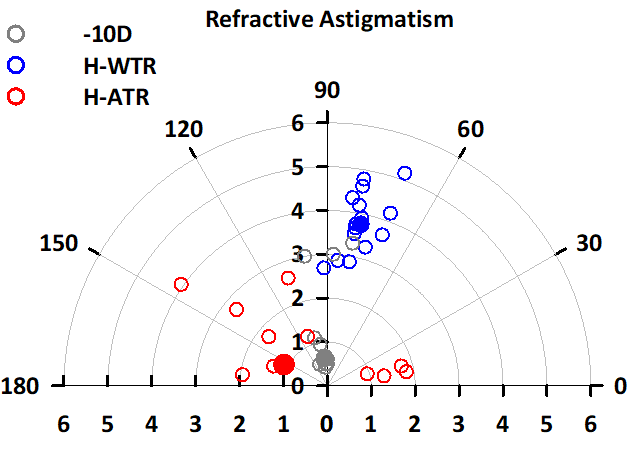


Supplementary Figure 2 Polar plot of refractive astigmatism induced by the three treatment groups.

The distribution of refractive astigmatisms obtained from the treated eyes of -10D (open grey circle), H-WTR (-6.00DS/-8.00DC x 90; open blue circle), and H-ATR groups (-6.00DS/-8.00DC x 180; open red circle) after one week of treatment (P5-P12). Each symbol represents the magnitude of astigmatism (distance from origin) and the axis of minus cylinder (degree) for one animal. Filled symbols represent the average refractive astigmatism for each group. At the end of the treatment period, both -10D and H-WTR groups developed astigmatism with axes near 90°, whereas H-ATR group developed astigmatism with axes near 180°.
